# Modeling of Unoriented Dendritic Grain Structures in Hard–Soft Magnetic Composites

**DOI:** 10.3390/ma19122547

**Published:** 2026-06-12

**Authors:** Grzegorz Ziółkowski

**Affiliations:** Institute of Materials Engineering, University of Silesia in Katowice, 75 Pułku Piechoty 1A, 41-500 Chorzów, Poland; grzegorz.ziolkowski@us.edu.pl

**Keywords:** spring-exchange magnetic composites, Monte Carlo simulations, magnetization processes, fractal-like systems, permanent magnets

## Abstract

This paper investigates the magnetization reversal processes in spring-exchange magnetic composites featuring irregular, dendritic structures. A disorder-based cluster Monte Carlo method combined with a Diffusion-Limited Aggregation (DLA) algorithm was used to model a fractal-like soft magnetic phase (Fe) embedded in a high-coercivity hard matrix (Fe-Nb-B-Dy). A multiparameter analysis was performed by varying the soft phase volume fraction (10–30%), intergrain exchange coupling via contact bridges (25–100%), system scale factors (1–20), surface-to-volume anisotropy ratios (*K_S_*/*K_V_* = 1–20), and the degree of random anisotropy contribution (*RAC* = 0–100%). The simulations reveal that highly branched fractal structures enhance the interfacial contact area, which accelerates the nucleation of domain reversal driven by the soft phase, paradoxically lowering the overall coercivity compared to compact morphologies. Furthermore, a lack of easy magnetization axis coherent alignment triggers a cascading reversal mechanism through local “weak links”, severely degrading the coercive field from approximately 4.2 T to below 0.4 T in extreme cases (at 30% Fe, 25% coupling and high *K_S_*/*K_V_* ratio). These findings suggest potentially the most important factors and their impact that should be taken into account in the design and optimization of next-generation powder-sintered permanent magnets.

## 1. Introduction

Hard magnetic materials with high coercivity play a central role in modern technologies, ranging from automotive and power engineering to sensors, actuators, and advanced automation systems. The highest-performance permanent magnets, based on compounds with a high content of rare-earth (RE) elements such as Nd and Dy, achieve $|B{H|}_{max}>\;400\;kJ/m^{3}$ [1,2]; however, this comes at the expense of high material cost, limited thermal stability, and significant geopolitical and environmental concerns. Consequently, intensive research efforts are focused on reducing the RE content while preserving high magnetic performance.

Current research directions can be divided into three main groups. The first comprises RE-free systems, such as Alnico [3,4,5,6,7], MnAl(C) [8,9,10], α-MnBi [11,12,13,14,15], α″-Fe_16_N_2_ [16,17,18,19,20], and ${L1}_{0}$-type phases (FePt [21,22,23], FeCo [24,25,26]), which are cheaper and more supply-stable but exhibit limited coercivity or remanence and, consequently, relatively low maximum energy product $|BH{|}_{max}$ (typically below ${100\;kJ/m}^{3}$). The second group includes conventional RE-Fe-B and RE-Co systems, which form the basis of commercial permanent magnets and achieve high $|BH{|}_{max}$ values or ultra-high coercivity (>7–9 T) [27,28,29], but are burdened by high cost, trade-offs among magnetic parameters, and intrinsic limitations such as a moderate Curie temperature (e.g., ~585 K for ${Nd}_{2}{Fe}_{14}$B) [30]. This indicates a fundamental limitation in the simultaneous maximization of coercivity and remanence within a single phase.

For the best currently available ${Nd}_{2}{Fe}_{14}$B-based magnets, the theoretical maximum $|BH{|}_{max}$, assuming an ideally rectangular hysteresis loop, is approximately ${510\;kJ/m}^{3}$ and follows directly from the relation $|B{H|}_{max}=\mu_{0}M_{s}^{2}/4$, where $M_{s}\;$ denotes the saturation magnetization [31,32]. In practice, the best commercially available magnets (e.g., N52) reach a maximum of approximately ${410\;kJ/m}^{3}$ but typically much less in high-temperature or high-coercivity variants containing Tb/Dy additions. For example, in the case of Nd–Fe–B type alloys with the main addition of Dy, such as Nd–Fe–B–Dy–Ga–Co, $|B{H|}_{max}$ ≈ 318 kJ/m^3^ is obtained while maintaining temperature stability up to about 150 °C [33]. The discrepancy between theory and practice results from microstructural factors such as imperfect texture, angular dispersion of the easy magnetization axis, the nature of grain boundaries, Nd-rich phases, porosity, defects, and grain inhomogeneity, all of which promote reverse-domain nucleation and reduce $\mu_{0}H_{c}$. Even small structural deviations can significantly reduce $|B{H|}_{max}$, which is why understanding these effects is essential.

Against this background, spring-exchange composites represent a promising alternative route. These systems offer a beneficial combination of soft and hard phase properties, merging the best characteristics of both material types into a single composite. Exchange coupling between a soft magnetic phase (high $M_{s}$) and a hard phase (large anisotropy) can lead to quasi-single-phase magnetization reversal and an increase in $|BH{|}_{max}\;$ relative to the individual constituents. Simulations indicate that values of ${200–400\;kJ/m}^{3}$ may be achieved with a significantly reduced RE content or even without RE elements in selected systems [34]. The high coercivity of the hard phase compensates for the reduction caused by the soft phase, making materials with ultra-high coercivity particularly advantageous [28]. However, simplified simulations often fail to fully reproduce the actual behavior of spring-exchange composites due to the complexity of the underlying interactions.

In this context, modeling of spring-exchange systems is highly important because of the complexity of these systems and the variety of properties that determine their magnetic characteristics. Such studies have already been performed for magnetization processes across a broad range of systems, from simple two-layer models [34] to fractal-like morphologies [35,36], which mimic structures formed by layer deposition or dendritic grain growth. Another important factor is the direction of the easy magnetization axes, that is, their distribution relative to the applied external magnetic field [37]. The results show numerous factors related both to composition and structure that must be considered in the design of spring-exchange magnets. However, previous analyses were usually limited and focused on isolated effects, whereas their interdependence is of decisive importance. Therefore, multiparameter studies are essential, particularly for systems close to real powder-sintered materials, such as the previously mentioned so-called neodymium magnets.

The main aim of this work is to investigate magnetization processes in spring-exchange magnetic composites containing magnetically soft (SM) and high- or ultra-high-coercivity hard magnetic (HM) phases. The simulation object consists of a fractal-like SM particle embedded in a “hard” magnetic matrix, itself built from an agglomerate of magnetically interacting HM grains, which can be regarded as a model of powder-sintered materials. The disorder-based cluster Monte Carlo method [38], which enables the analysis of multiphase magnetic systems, was used as the simulation tool. The study focuses on a comprehensive analysis that simultaneously considers the following factors: (i) degree of fractal development, (ii) content of the soft magnetic phase, (iii) magnetic coupling between all grains, (iv) total system size, (v) surface-to-volume anisotropy ratio, and (vi) ordering of the easy magnetization axes of the HM grains.

## 2. Systems and Simulation Procedure

The system construction procedure consists of four distinct phases. In the first phase, the target system is empty and divided into 12 segments along each of the three spatial dimensions, resulting in a total of 1728 initially vacant segments, where future magnetic grains may be placed. Within this discretized system, a seed is introduced at the center (occupying the first grain), and its growth is then guided by the Diffusion-Limited Aggregation (DLA) algorithm.

The DLA algorithm operates by iteratively repeating three steps:

(a) Introducing a new element into the system, starting from a randomly chosen boundary (a so-called “particle” in the context of the DLA procedure);
(b) Allowing the “particle” to undergo a random walk until it attaches to the existing seed (or growing fractal structure); and
(c) Fixing the “particle” in place upon attachment to the system.

**Figure 1:**
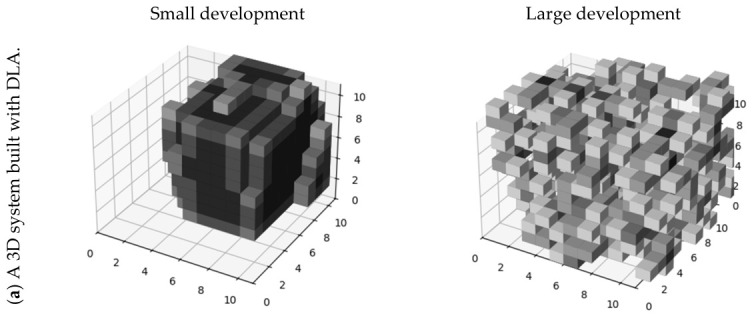

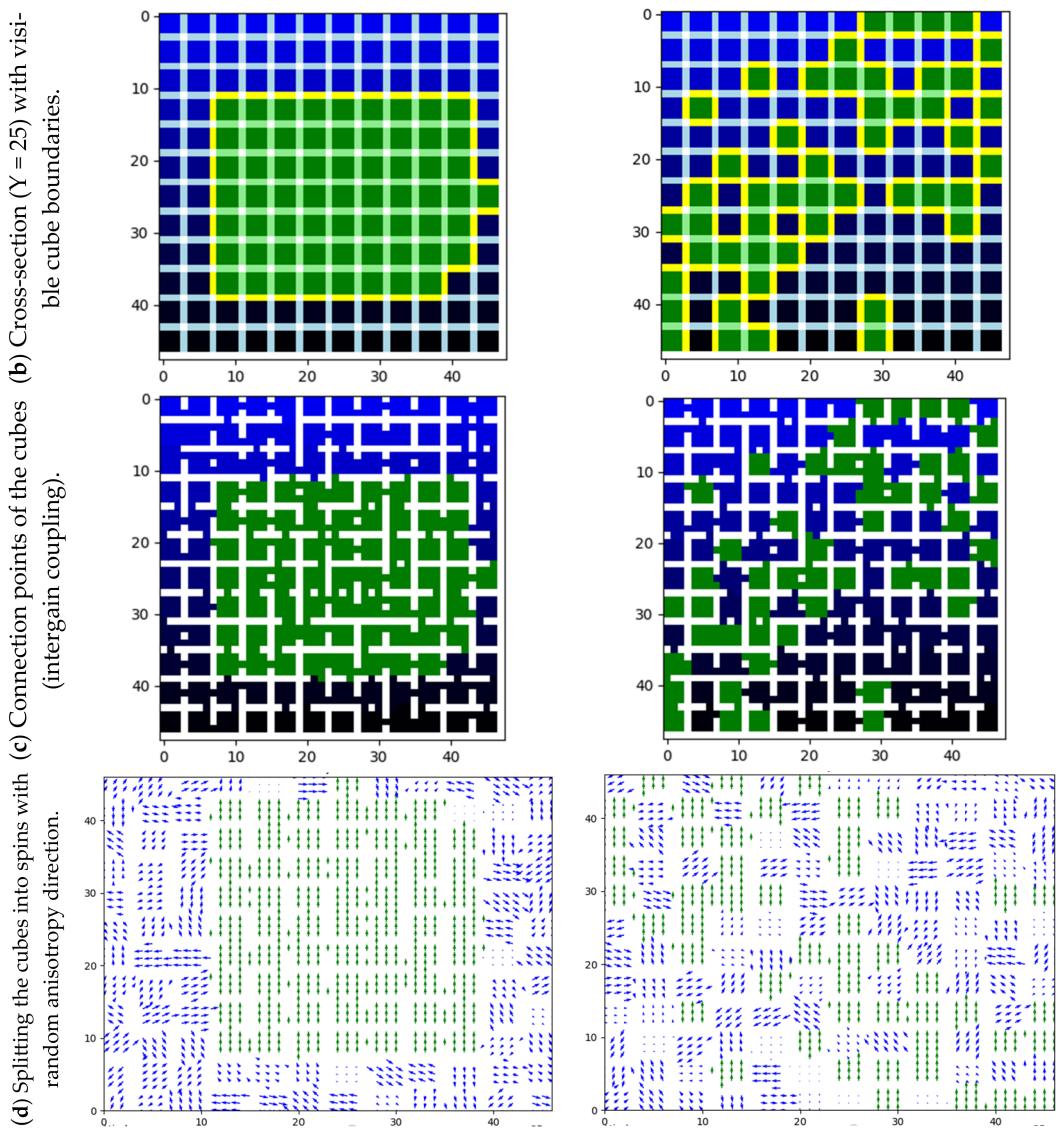
A matrix of figures showing examples of simulated systems with low (**left**) and high (**right**) structural complexity—demonstration of the four phases of system construction: (**a**) fractal structure of the soft phase generated using the DLA algorithm; (**b**) surrounding the soft magnetic core (green) with hard-phase grains (blue); (**c**) introduction of a bridging parameter to regulate exchange coupling between grains; (**d**) representation of each grain by a 3 × 3 × 3 spin lattice with realistic physical parameters, resulting in a total system size of 48 × 48 × 48 nodes (cross-section shown).

This procedure leads to the emergence of fractal-like structures characteristic of dendritic grain growth. Furthermore, the process can be regulated by parameters such as the probability of attachment upon collision with another “particle”, enabling the formation of either more compact (low attachment probability) or more highly branched (high attachment probability) structures. Figure 1a shows the system at the end of the first phase of development for both cases. For visual clarity, each segment is shaded according to the number of neighboring segments it has (darker shades indicate higher neighborhood density).

The degree of structural complexity obtained clearly correlates with the surface-to-volume ratio, which is expected to enhance the influence of surface anisotropy and, consequently, affect the magnetization process.

The structure obtained in the first phase corresponds to an agglomerate of grains of the soft magnetic phase (e.g., produced by powder sintering), where each segment represents a single grain. Since the aim of this study is to investigate magnetization processes in composites comprising both soft and hard magnetic phases, the second stage of system construction involves filling the space around the soft-phase structure with grains of the hard magnetic phase. Figure 1b illustrates a cross-section of the resulting system. The green and blue colors correspond to the soft and hard magnetic phases, respectively. Additionally, the boundaries of individual grains are marked in light blue, while the interface between the two phases is highlighted in yellow.

In real systems, individual grains are unlikely to be perfectly bonded to one another, which leads to a weakening of magnetic coupling at the grain boundaries. This is a key factor influencing the magnetization process in spring-exchange systems and cannot be neglected. To reflect this condition in the simulated structures, the third stage involves separating individual grains—both soft and hard magnetic—while preserving only a subset of direct contact points, referred to as bridges. The number of bridges is a controllable parameter, while their distribution is randomly assigned with uniform probability. This bridge-count parameter directly controls the strength of magnetic coupling between grains and is expressed as a percentage: 100% corresponds to ideal grain–grain contact, while 0% denotes a complete absence of bridges. An example of the structure after completion of the third phase is shown in Figure 1c.

The final step in the system preparation involves transforming each grain into a set of magnetic spins used in the simulation. Each grain is approximated as a 3 × 3 × 3 array of spins, with an additional one-spin-wide gap between adjacent grains, unless a bridge is present at that location. As a result, the final system consists of 48 × 48 × 48 magnetic spins (nodes). The fully transformed system is illustrated in Figure 1d.

At this stage, each spin is assigned physical parameters including the spin *S*, exchange integral *J*, magnetic anisotropy *K*, and the direction of the easy magnetization axis. These values correspond to either the soft (Fe) or hard (Fe-Nb-B-Dy) magnetic phase, as specified in Table 1.

A key factor is the assignment of the easy magnetization axis direction for each grain. In real systems, depending on the processing and alignment method used—such as sintering in the presence of an external magnetic field—grains may exhibit varying degrees of alignment along a common axis. In particular, if no alignment methods are applied, the orientation of the easy magnetization axis may be completely random. One of the objectives of this study is to investigate the effect of this phenomenon on the magnetization behavior and related parameters such as coercivity. Therefore, in the final construction stage, each hard-phase grain is assigned either an aligned easy magnetization axis (along the direction of the external magnetic field) or a randomly oriented one. This direction is consistent for all spins within a given grain. The fraction of hard-phase grains with randomly oriented easy magnetization axes is controlled by a dedicated parameter (*RAC*). Example directions assigned to individual spins are shown in Figure 1d.

Rational modeling of magnetization processes in such complex, multiphase systems requires advanced computational tools, among which Monte Carlo techniques, and specifically cluster Monte Carlo methods, play a central role by enabling simulations at both zero and finite temperatures. To accurately modeling of hysteresis loops in spring-exchange systems with irregular geometries, a specialized disorder-based Monte Carlo approach has been implemented [38], further enhanced by dedicated scaling rules for system parameters and procedures to overcome hardware-related node count limitations [39]. This approach consists in introducing an original extension based on the configuration entropy of properties such as magnetic anisotropy, significantly improving the efficiency of locating free-energy minima in complex nanostructures and fractal-like morphologies. Consequently, this integrated approach provides a powerful platform for systematically exploring the interplay between microstructure, intergrain coupling, and magnetic properties, paving the way toward the design of next-generation spring-exchange permanent magnets.

Using this method, a total of 672 magnetization curve were simulated for external magnetic field values ranging from 0 to −7 T. The study focused on the following areas and parameters:

(a) **Degree of fractal development**, i.e., Fe surface-to-volume ratio—All simulations included two representative cases. These corresponded to the least and most developed configurations of the soft-phase grain agglomerate achievable within the defined system setup.
(b) **Content of the soft magnetic phase** in the overall composite—This parameter, denoted as *Fe*, was varied as a percentage of total volume: 10%, 20%, and 30%.
(c) **Magnetic coupling between all grains** of both soft and hard phases—This was controlled by the number of bridges, expressed as the percentage of inter-grain contact retained: 25%, 75%, and 100%. The exchange integral value assigned to each bridge matched the nearest grain type. A coupling level of 100% was used only in conjunction with the highest soft-phase content (30%).
(d) **Overall system size**, controlled using a scaling factor (1, 10, 20) in accordance with the developed scaling rules.
(e) **Surface-to-volume anisotropy ratio** at the boundary of each grain, with considered values of 1, 5, 10, and 20.
(f) **Ordering of easy magnetization axes** in magnetic grains, controlled by a percentage parameter *RAC* (Random Anisotropy Contribution), which defines the fraction of hard-phase grains with randomly oriented easy axes.

All variable as well as constant parameters of the system and simulation are summarized in Table 1.

## 3. Results

**Figure 2:**
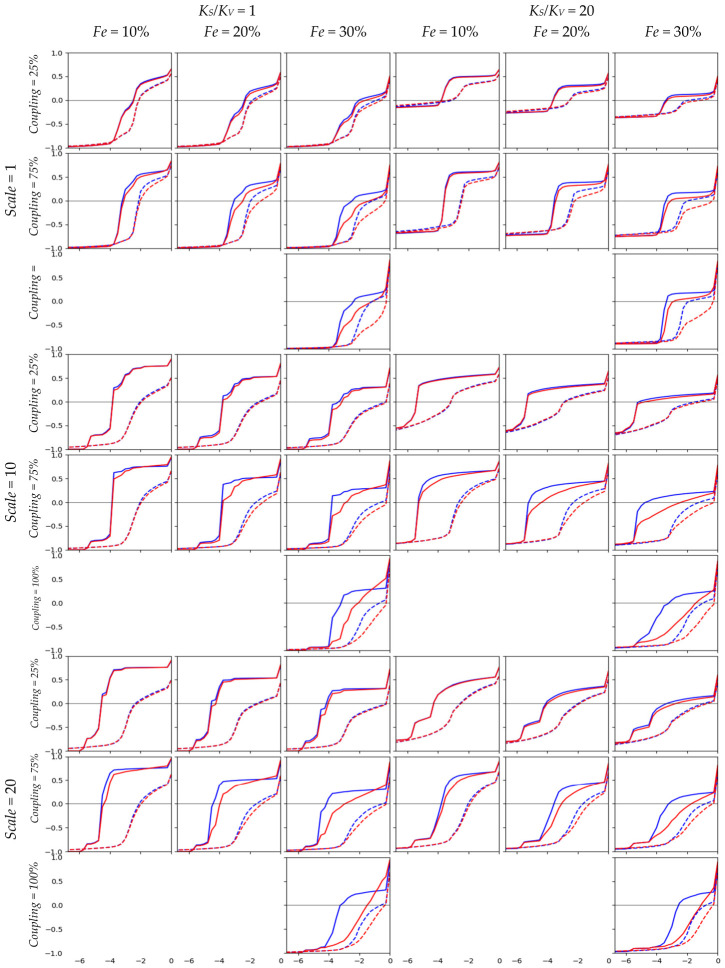
Demagnetization curves for low (blue)- and high (red)-degree-of-development systems for systems with ordered (continuous line) and disordered (dashed line) grain easy magnetization axis.

Figure 2 presents the raw magnetization reversal curves obtained for selected systems with different values of magnetic coupling, iron content, and system scale. For clarity, only two limiting cases of the surface-to-volume anisotropy ratio are shown: the case in which both anisotropy contributions are equal and the case in which the surface anisotropy is twenty times larger than the volume anisotropy. Each plot compares magnetization curves for systems characterized by a low and a high degree of fractal development of the soft-phase grain agglomerate. These are represented by the blue curve (low fractal development) and the red curve (high fractal development), respectively. Examples of the corresponding fractal-like structures are shown in Figure 1a.

An additional aspect considered in the analysis is the parameter *RAC* (Random Anisotropy Contribution), which describes the degree of alignment of the easy magnetization axes in hard-phase grains. Solid lines correspond to systems in which the easy axes of all grains are perfectly aligned along a single direction, which is the most favorable from the perspective of hard magnetic properties (*RAC* = 0%). In contrast, dashed lines represent systems in which the direction of the easy magnetization axis for each grain is assigned independently at random (*RAC* = 100%).

The analysis of the obtained data reveals several important trends. Systems with disordered easy magnetization axes exhibit significantly lower coercivity compared with systems in which the axes are aligned. This effect becomes increasingly pronounced with increasing system size. In extreme cases, the coercive field may decrease from values close to 4.5 T to below 0.5 T, for example, for a system containing 30% of the iron phase, weak intergrain coupling (25%), and a high surface anisotropy contribution (*Ks*/*Kv* = 20). The magnitude of this change is also correlated with the content of the soft magnetic phase. Therefore, it is instructive to analyze more broadly intermediate values of the *RAC* parameter as well, which will be discussed in the following section of the paper.

Furthermore, in all simulated cases, systems characterized by a higher degree of fractal development exhibited comparable or noticeably lower coercivity values than systems with a more compact structure. This effect depends on both the iron-phase content and the strength of intergrain coupling, with its magnitude increasing as these parameters grow. The overall system size (related to the scaling factor) also plays an important role—as the system size increases, the differences between the considered configurations become increasingly pronounced.

**Figure 3:**
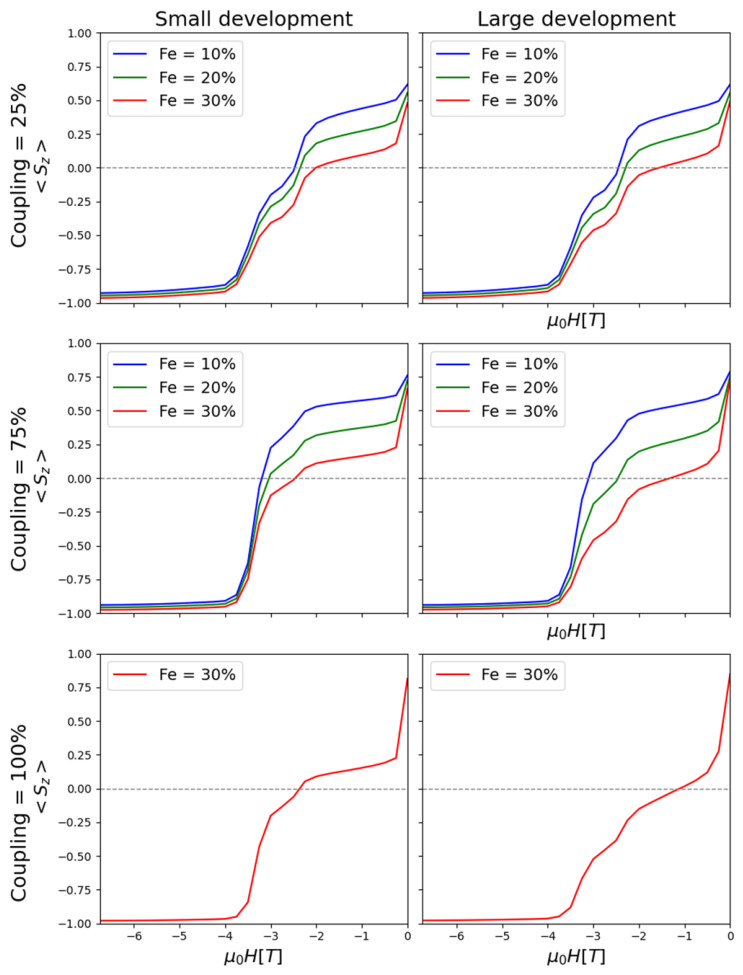
Remagnetization curves (separately for systems with small (on the left) and large (on the right) degree of grain development) illustrating the influence of the soft phase content and the intergrain coupling. For all illustrated cases, *scale* = 1, *K_S_*/*K_V_* = 1, *RAC* = 0.

As expected, the iron content plays a crucial role in maintaining hard magnetic properties. A detailed analysis of this aspect is presented in the plots shown in Figure 3. For clarity, the discussion at this stage is restricted to systems with fully aligned easy magnetization axes (*RAC* = 0%), the smallest considered system scale, and an equal ratio of surface-to-volume anisotropy contributions.

As can be observed, the coercive field decreases systematically with increasing Fe content. For example, in the system with a coupling level of 75%, the coercivity decreases from approximately 3.2 T to about 2.5 T as the Fe content increases from 10% to 30%. This reduction is even more pronounced for the system with a more developed fractal structure, where at 30% Fe the coercivity decreases by approximately 1.2 T. Similar differences are observed even for ideal coupling (100%), where the coercivity of the more developed system at 30% Fe decreases further, reaching values of about 1 T. These results suggest that the reduction in coercivity becomes more pronounced in systems with more developed fractal structures and stronger intergrain coupling, which may appear counterintuitive.

It is also worth noting that, in all analyzed cases, the magnetization reversal curves exhibit an irregular shape, indicating a gradual reversal of magnetization in different regions of the system. The rapid drop in magnetization observed immediately after the external magnetic field crosses zero can be associated with the reversal of the magnetically soft iron phase. Consequently, this effect becomes more pronounced with increasing Fe content. This initial process is followed by a gradual magnetization reversal of the hard magnetic phase.

**Figure 4:**
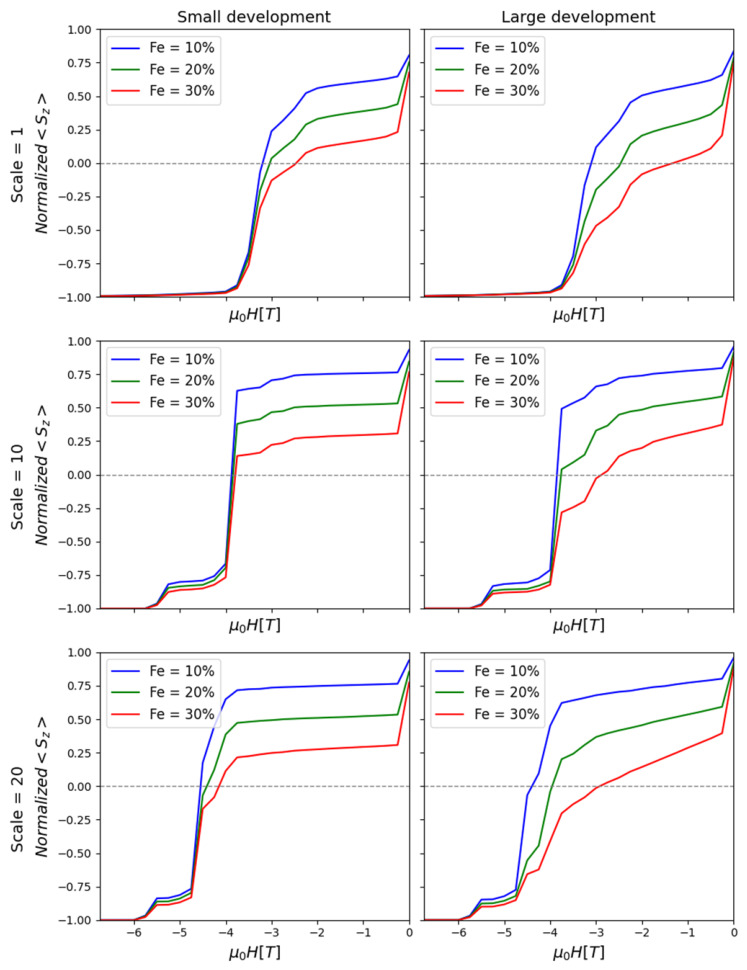
Remagnetization curves (separately for systems with small (on the left) and large (on the right) degree of grain development) illustrating the influence of the soft phase content and the system size (*scale*). For all illustrated cases, coupling = 75%, *K_S_*/*K_V_* = 1, *RAC* = 0.

The analysis of the influence of iron content is complemented by the results presented in Figure 4. In this case, the focus is placed on the effect of system scale, while the intergrain coupling level is fixed at 75%. As can be observed, larger systems exhibit significantly higher coercivity values. For example, for Fe = 10% the coercive field increases from approximately 3.2 T, through 3.9 T, to more than 4.5 T for scaling factors of 1, 10, and 20, respectively. A similar trend is also observed for systems with higher Fe content and for structures with a greater degree of fractal development, although the maximum coercivity values remain correspondingly lower, as discussed earlier. Moreover, the magnetization reversal curves for the more strongly scaled systems exhibit a more square-like shape, suggesting a more coherent resistance to magnetization reversal originating from the hard magnetic phase. At the same time, the magnetically soft iron phase undergoes rapid reversal, consistent with the behavior described in the previous cases.

**Figure 5:**
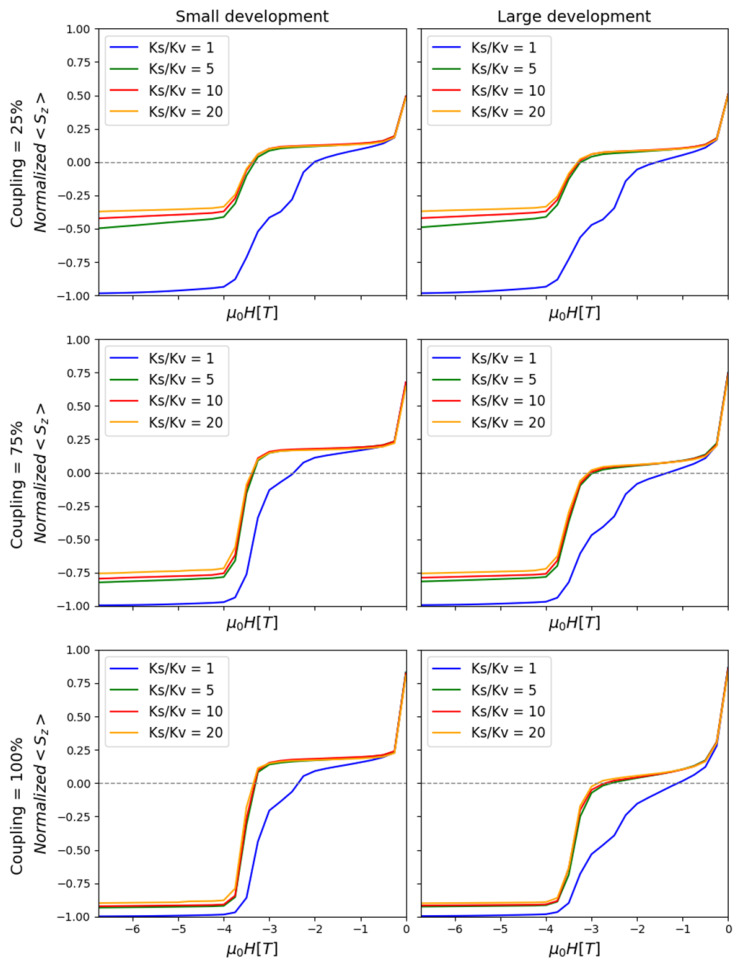
Remagnetization curves (separately for systems with small (on the left) and large (on the right) degree of grain development) illustrating the influence of the surface to volume anisotropy ratio, as well as the intergrain coupling. For all illustrated cases, *scale* = 1, *Fe* = 30%, *RAC* = 0.

**Figure 6:**
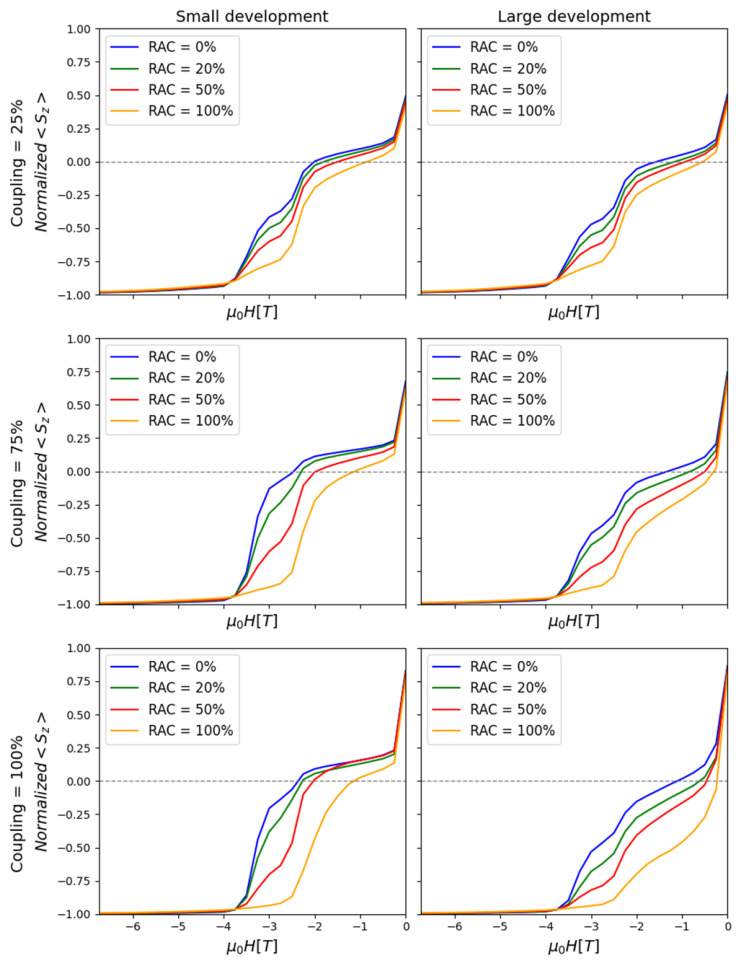
Remagnetization curves (separately for systems with small (on the left) and large (on the right) degree of grain development) illustrating the influence of the random anisotropy contribution (*RAC*), as well as the intergain coupling. For all illustrated cases, *scale* = 1, *K_S_*/*K_V_* = 1, *Fe* = 30%.

The plots presented in Figure 5 illustrate differences in the magnetization reversal curves for systems characterized by different ratios of surface-to-volume anisotropy. In turn, Figure 6 focuses on the comparison of systems with different degrees of alignment of the easy magnetization axes, whereas the previous analysis considered only fully ordered systems. In both figures, the system scale and iron content were fixed at 1 and 30%, respectively.

As expected, systems with a higher contribution of surface anisotropy, i.e., for which *K_S_*/*K_V_* > 1, exhibit higher coercivity values. This behavior results from the overall increase in the effective magnetic anisotropy of the system. In contrast, the comparison of magnetization curves for different values of the *RAC* parameter clearly demonstrates a deterioration of hard magnetic properties with increasing disorder in the orientation of the easy magnetization axes. More detailed comparisons of the results (not included in this paper) indicate that increasing disorder leads not only to a reduction in coercivity but also to decreases in saturation magnetization and magnetic remanence. This effect was observed for all analyzed systems and progressed approximately linearly with increasing *RAC*.

## 4. Discussion

**Figure 7:**
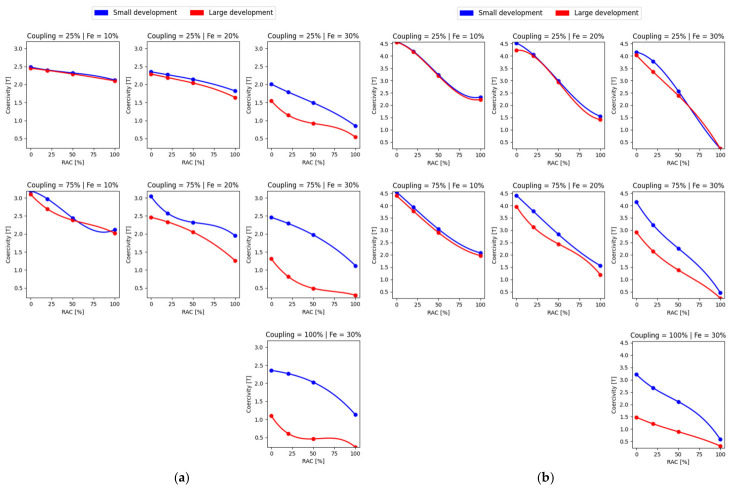
Summary of the influence of random anisotropy contribution (*RAC*), as well as the system size (*scale*), soft phase content and intergrain coupling, on the coercivity. For all illustrated cases, the surface to volume anisotropy ratio is equal to 1. (**a**) *Scale* = 1. (**b**) *Scale* = 20.

A summary of all observed trends in the variation in coercivity, additionally presented as a function of the degree of randomness in the orientation of the easy magnetization axes, is shown in Figure 7. The analysis of the magnetization curves reveals the expected decrease in coercivity with increasing iron content. However, a particularly notable feature is the clear difference between systems characterized by different degrees of fractal development. In all analyzed cases, systems with a more developed fractal grain structure exhibit lower (or similar) coercivity than systems with a more compact morphology, characterized by a higher density of Fe grains and a lower degree of branching.

Moreover, this difference increases not only with increasing iron content but also with increasing strength of exchange interactions (i.e., the number of bridges between grains) as well as with increasing randomness in the orientation of the easy magnetization axes. Such behavior is not immediately intuitive and may appear counterintuitive. A more highly branched structure is expected to exhibit stronger shape and surface anisotropy, as well as a higher surface-to-volume ratio, which would normally favor stronger coupling between the grains and, consequently, an improvement in hard magnetic properties.

The origin of the observed behavior may be attributed to two complementary mechanisms. First, the rapid decrease in magnetization already at relatively low external magnetic fields (see Figure 2, Figure 3, Figure 4, Figure 5 and Figure 6) indicates significant reversal of the magnetically soft phase. Through exchange interactions, this phase can subsequently initiate and accelerate the magnetization reversal of the hard phase. In this context, increasing the intergrain coupling—either by introducing a larger number of bridges between grains or by increasing the surface-to-volume ratio (i.e., by enhancing the fractal development of the Fe grain structure)—strengthens the influence of the soft phase on the hard phase and promotes a faster reversal of its magnetization.

The second possible mechanism is related to the greater diversification of the local environment of hard-phase grains in structures with a higher degree of fractal development. Under such conditions, the magnetization reversal process may initiate at lower magnetic field in grains that are more weakly coupled to the rest of the hard phase and are more strongly surrounded by the soft phase. Once reversed, each such hard-phase grain facilitates, via exchange interactions, the reversal of neighboring grains, leading to a gradual propagation of the magnetization reversal process. This mechanism contrasts with the more coherent rotation of magnetic moments expected in systems with a more compact structure, where the absence of such “weak links” requires a higher external field to trigger reversal.

This effect is particularly evident when comparing the magnetization curves for systems with different degrees of fractal development but similar Fe content (for example, Figure 4 with scale = 20 and 30% of iron). Furthermore, increasing the fraction of grains with randomly oriented easy magnetization axes (parameter *RAC*) significantly enhances the described mechanism, as it increases the probability of the occurrence of such “weak links”, whose reversal is facilitated by their different orientation with respect to the saturation direction of the system. As a result, the magnetization reversal process begins at lower magnetic fields and proceeds faster, which is especially evident for the more developed structures (red curves) in Figure 7a with 30% of Fe.

**Figure 8:**
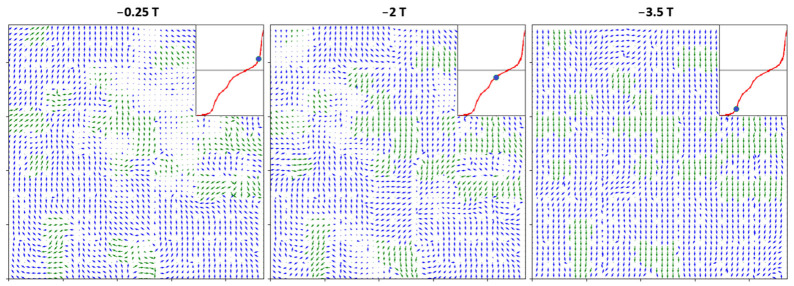
Cross-sectional view of a system containing 30% iron phase with a highly developed fractal structure, maximal coupling, *RAC* = 0%, *scale* = 1 and *K_S_*/*K_V_* = 1. Green denotes the iron phase, while blue represents the magnetically hard phase. Arrows indicate spin directions. The panels correspond to different stages of the magnetization process under an external magnetic field.

The described phenomenon can be directly observed by analyzing the evolution of spin configurations during the magnetization process, as shown in Figure 8. The three panels present cross-sections of the same system under an increasing external magnetic field, ranging from −0.25 T to −3.5 T. The considered case corresponds to a system containing 30% iron phase, characterized by a highly developed fractal structure, fully ordered easy-axis orientation, maximal (100%) intergranular coupling, no system scaling, and a uniform ratio of surface to volume anisotropy. For comparison, the corresponding hysteresis curve is shown in Figure 2 (third panel from the top and third from the left, solid red line). In Figure 8, the iron phase is marked in green and the magnetically hard phase in blue, while the vectors represent spin directions. As can be observed, even at very weak magnetic fields, nearly the entire soft phase undergoes remagnetization, gradually inducing reorientation of the hard phase spins in the neighborhood. At approximately −2 T, about half of the hard phase is remagnetized, while at −3.5 T the system approaches a nearly fully saturated state. A similar behavior is observed across all analyzed systems.

**Figure 9:**
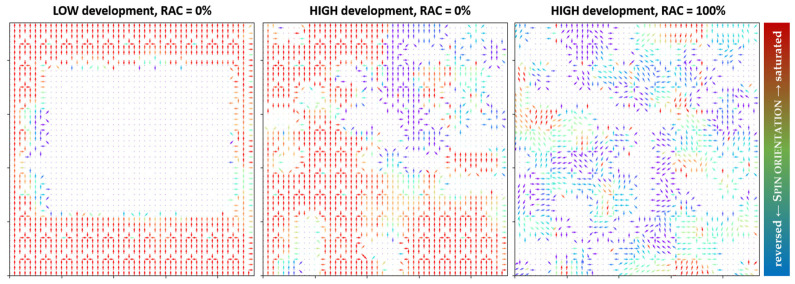
Distribution of the anisotropy axis vectors of the hard magnetic phase (cross-section) for three different types of systems. Vectors indicate the direction of the easy magnetization axis (absence denotes the soft phase), while the color scale represents the spin orientation, i.e., the degree of remagnetization (red—initial state, blue—fully remagnetized). All systems are shown under an external magnetic field of −0.5 T.

Figure 9 enables an analysis of the properties of spins that undergo the fastest remagnetization, while simultaneously accounting for their local environment and the orientation of the easy magnetization axis. Note that, in contrast to Figure 8, the vector shown in this illustration directly indicates the direction of the easy magnetization axis (in particular, the absence of a vector corresponds to zero anisotropy, i.e., the soft phase), whereas the color scale reflects the spin orientation, i.e., the degree of remagnetization. Red denotes preservation of the initial orientation, while blue indicates complete remagnetization.

All three panels depict the state of the system under the same external magnetic field of −0.5 T, but for three distinct structural configurations: a low-development fractal with an ordered easy-axis orientation (where deviations occur primarily at grain boundaries and are associated with surface anisotropy) and two systems containing a highly developed fractal, differing in the *RAC* parameter. Comparative analysis reveals three key aspects. First, in the low-development fractal, remagnetization of the hard magnetic phase occurs predominantly in spins directly adjacent to the soft phase. Owing to the relatively small surface area of this phase (visible as an empty region), its influence on the hard phase is limited, and the remagnetization process proceeds gradually without triggering an abrupt transformation of the entire system. Second, for the highly developed fractal (middle panel), the significantly larger surface area with the soft phase leads to the simultaneous remagnetization of a large number of neighboring spins, initiating a collective effect and resulting in a more abrupt system response (see Figure 8). Third, when additional disorder in the easy-axis orientation is introduced (right panel), nearly complete remagnetization of the system is observed despite the same external field as in the previous cases. This process is particularly pronounced for spins whose easy axes are substantially misaligned with the direction of the applied magnetic field, which in this case corresponds to the vertical orientation. The reduced energy barrier associated with the anisotropy direction facilitates early-stage remagnetization of a large population of spins, effectively creating a “critical mass” that drives a cascade remagnetization of the entire system.

In summary, all the discussed aspects indicate that the key factor determining the overall behavior of the system and its susceptibility to rapid magnetization reversal is the structural configuration, particularly the immediate environment of the soft phase and the extent of the interfacial contact area. At the same time, factors such as strong magnetic coupling and incomplete alignment of the easy magnetization axes of individual grains most significantly amplify this effect.

## 5. Conclusions

The main objective of this work was to investigate, using simulations of magnetization processes in systems with complex structures, the factors affecting the deterioration of hard magnetic properties with respect to their theoretical values. The analysis was carried out taking into account multiple structural and magnetic aspects, as well as their mutual interdependencies.

The applied simulation approach—the disorder-based Cluster Monte Carlo method combined with algorithms generating branched geometries (like DLA)—can be effectively used to analyze magnetization processes in composites composed of magnetically hard and soft phases. The obtained results reveal the complexity of mechanisms governing the magnetization reversal process and highlight the significant role of system geometry.

The main conclusions from the analyses carried out can be summarized as follows.

➢ **Influence of grain structure development on magnetic properties:** It has been demonstrated that the degree of grain structure development may, depending on the combination of other parameters, substantially modify (in particular, deteriorate) the hard magnetic properties of the system. Consequently, the increased surface anisotropy characteristic of more branched structures is not the only factor determining the magnetic behavior of such composites. For instance, in extreme cases (e.g., 30% Fe, 100% coupling and 0% RAC), a decrease in coercivity from about 2.4 T to 1.1 T was observed.
➢ **Dominant role of the soft magnetic phase in magnetization reversal:** In the analyzed systems, the magnetically soft phase plays the dominant role, undergoing rapid magnetization reversal already at relatively low external magnetic fields. Once reversed, these grains accelerate the reversal of the hard magnetic phase through exchange interactions.
➢ **Factors accelerating reversal of the hard magnetic phase:** Although the content of the soft phase plays a dominant role in initiating the magnetization reversal process, factors such as (i) stronger intergrain coupling, (ii) increased interfacial contact area resulting from a more developed (branched) grain geometry, and (iii) poor alignment of the hard phase easy magnetization axes accelerate this process and lead to a magnetization reversal of the magnetically hard phase in a lower magnetic field.
➢ **Non-uniform easy-axis alignment as a degradation mechanism:** A particularly important factor deteriorating the hard magnetic properties is the non-uniform alignment of easy magnetization axes in individual grains. In combination with the mechanism described above, a non-optimal orientation of the easy axis in a single grain facilitates its reversal. Such a reversed “weak link” subsequently promotes the reversal of neighboring grains through exchange interactions, triggering a cascading magnetization reversal throughout the system. For instance, in extreme cases (at 30% Fe, 25% coupling and *K_S_*/*K_V_* = 20), the coercive field drops more than tenfold, from approximately 4.2 T (full coherent alignment) to below 0.4 T (complete lack of coherent alignment).

## Figures and Tables

**Table 1:** The main parameters for all simulated systems and their values.

Parameter	Designation	Values
Soft phase content	*Fe*	10%, 20%, 30%
Coupling between the grains	*coupling*	25%, 75% (and 100% for *Fe* = 30%)
System size (scale factor)	*scale*	1, 10, 20
Surface to volume anisotropy ratio	*K_S_*/*K_V_*	1, 5, 10, 20
Random anisotropy contribution	*RAC*	0%, 20%, 50%, 100%
Fractal development	*development*	small, large
Spin for soft/hard magnetic phase	*S_S_*, *S_H_*	1.1, 0.93
Anisotropy for soft/hard phase	*K_S_*, *K_H_*	0 eV, 3.45 × 10^−4^ eV
Exchange integral for soft/hard phase	*J_S_*, *J_H_*	1.23 × 10^−2^ eV, 9.8 × 10^−3^ eV
System size (number of nodes)	*N*	48 × 48 × 48

## Data Availability

The original contributions presented in this study are included in the article. Further inquiries can be directed to the corresponding author.
